# Interaction between repetition suppression in motor activation and long-interval intracortical inhibition

**DOI:** 10.1038/s41598-019-47932-9

**Published:** 2019-08-08

**Authors:** Shohreh Kariminezhad, Jari Karhu, Laura Säisänen, Mervi Könönen, Petro Julkunen

**Affiliations:** 1 Department of Applied Physics, University of Eastern Finland, Kuopio, Finland; 2 Department of Clinical Neurophysiology, Kuopio University Hospital, Kuopio, Finland; 3 Nexstim Plc, Helsinki, Finland; 4 Department of Clinical Radiology, Kuopio University Hospital, Kuopio, Finland

**Keywords:** Neuroscience, Neuronal physiology, Medical research

## Abstract

Repetition suppression (RS) is the adaptation of the neural activity in response to a repeated external stimulus. It has been proposed that RS occurs at the thalamo-cortical level, hence activating a feedback loop to the cortex in order to counteract with the repeated motor cortical activation. In this study, to elucidate the common modulators between the RS and the inhibitory/facilitatory cortical networks, two TMS paradigms were applied, i.e. the characteristic long-interval intracortical inhibition (LICI) and the I1-wave timed short-interval intracortical facilitation (SICF). Since LICI is a local intracortical inhibitory phenomenon affecting cortical excitation over a long interval like the RS, the interaction between RS and LICI was tested. As the I1-wave timed SICF is likely not affected by inhibitory modulation, the appearance of the RS with respect to SICF was investigated. Non-linear interaction between LICI and RS was observed, while I1-wave timed SICF facilitated all MEP responses of RS by a common offset still preserving the RS. These findings implicate that the underlying mechanism for the observed interaction is likely contributed to the activation of the negative thalamo-cortical feedback loop represented by the RS, most likely at the cortical level.

## Introduction

The primary motor cortex is a highly organized, five-layered convoluted sheet of neural cells, located on the precentral gyrus of the cerebral cortex. In the motor cortex, intermingled with the inhibitory GABAergic interneurons, the excitatory glutamatergic pyramidal neuron is the principal cell type^1,2^. Pyramidal cells are most abundant in layers III and V. However, their horizontal and vertical extensions into other layers provide the motor cortical networks with a flexible synaptic organization^3^. This organization provides a cortical platform for neuroplasticity in the primary functions, such as movements. Neuroplasticity is a crucial characteristic in the central nervous system, enabling recruitment of the neuronal connections to adapt, as well as to maladapt, to modified requirements. Neuroplasticity is also mediated by the inhibitory GABAergic interneurons^4^.

Transcranial magnetic stimulation (TMS) is a non-invasive technique that allows us to study the facilitatory and inhibitory cortical networks by means of time-varying magnetic fields^5^. TMS-evoked facilitation results in a number of cortico-spinal descending volleys in the pyramidal tracts: direct (D) and indirect (I) waves. D-waves, produced via direct activation of the pyramidal neurons of layer V, are the earliest of these descending volleys. While the origin of later I-waves is contributed to polysynaptic connections between the neurons in layer II/III and those in layer V, I1-wave is proposed to originate from the monosynaptic excitatory cortico-cortical projections on the cortico-spinal fibers^6^. Supporting the notion that the GABAergic inhibitory system is not involved in the formation of the I1-waves, insensitivity of these waves to GABA_A_ agonists has been demonstrated^7^. To characterize the I-waves, short-interval intracortical facilitation (SICF) is a well-documented paired-pulse TMS paradigm. There is convergent evidence that the facilitatory interaction of the paired pulses in SICF occurs primarily at the cortical level^8^. SICF has been conventionally demonstrated using monophasic waveform^8^. However, it has been recently evoked applying biphasic waveform^9,10^. Repetition suppression (RS) refers to the adaptation of the neural activity in response to repeated external stimuli^11,12^. The recovery of the habituated response after the heightened initial response implies that the RS functions to prevent the brain from overreaction to a novel stimulus^13^. Impairment of the RS has been demonstrated previously in progressive myoclonus epilepsy and schizophrenia^14,15^. It has been suggested the RS occurs at the thalamo-cortical level, following TMS^14,16^. This phenomenon has been demonstrated in the motor system as a decrement in the amplitudes of the subsequent motor evoked potentials (MEP) following the initial one^16,17^. The decline in the MEP amplitudes indicates the adaptive behavior of the motor system. Adaptation has been associated with neuroplastic changes in the nervous system^18^, and therefore as an objective biomarker to assess the neuroplastic abilities. Several mechanisms have been postulated regarding the neurophysiological basis of the inhibitory phenomenon observed in the RS^11^. One such underlying mechanism might contribute to the increased activity of GABAergic inhibitory system, a mechanism that is also known to mediate long-interval intracortical inhibition (LICI)^19,20^. LICI is an intracortical inhibitory phenomenon that is demonstrated as suppression of neural activity, when a suprathreshold stimulus is followed by a second suprathreshold stimulus at an inter-stimulus interval (ISI) of 50–200 ms^20^. The inhibition time course of LICI and the pharmacological studies reflect the cortical origin and activation of the GABA_B_ inhibitory receptors in this phenomenon^21,22^. In this study, we applied two stimulation paradigms with the RS: (1) SICF interaction in I1-wave and (2) LICI, to test the effects of immediate cortical facilitation and a cortical long-interval inhibitory modulation on the RS. The appearance of RS in relation to facilitatory I1-wave timed SICF, was applied as it is suggested to be unaffected by inhibitory modulation^23,24^, while LICI is known to be mediated by GABA_B_^25–27^. We hypothesized that I1-wave timed SICF would facilitate all MEP responses in the RS without affecting the inhibitory modulation, while the interaction of the LICI and RS was hypothesized to occur through the RS affecting the cortical inhibitory function through the thalamo-cortical feedback.

## Results

The typical main effect of RS was observed in RS-baseline paradigm (*F*(3,56) = 19.24, *p* = 0.004), where the second, third and fourth MEP amplitudes were significantly lower than the first one (*p* < 0.001, *p* = 0.001, *p* = 0.002 for normalized MEPs, respectively). No difference in MEP amplitude was observed between the second and third, third and fourth, and fourth and second stimuli (*p* > 0.1). Further, to show the inhibitory effect of LICI and facilitatory effect of SICF at baseline, a Wilcoxon signed-rank test revealed a significant inhibition, on average of 92%, induced by the LICI paradigm (*p* = 0.012), and a paired samples t-test indicated facilitation effect on MEPs (273% on average), induced by the SICF paradigm (*p* = 0.013).

**Figure 1:**
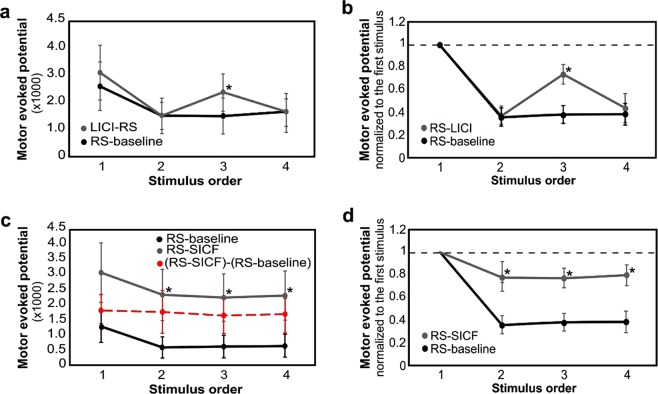
(**a**) Absolute MEP amplitudes and (**b**) normalized MEP amplitudes (*mean* ± *standard error*) across all participants within the trains during RS-baseline and RS-LICI paradigms. The third MEP amplitude in RS-LICI paradigm was significantly higher than that in the RS-baseline paradigm. (**c**) Absolute MEP amplitudes (*mean* ± *standard error*) across all participants within the trains during the RS-baseline and RS-SICF paradigms. MEPs in RS-SICF paradigm are increased by a common offset while the absolute RS effect remains unchanged. The dashed red line marks the MEPs of RS-SICF subtracted from those of RS-baseline. (**d**) Normalized MEP amplitudes (*mean* ± *standard error*) across all participants within the trains during RS-baseline and RS-SICF paradigms demonstrating an increase of the normalized MEPs in the RS-SICF paradigm. The MEPs were averaged over all trains. Normalization of MEPs were performed with respect to the mean MEP amplitude induced by the first stimulus (dashed line). Asterisk indicates significant differences for pairwise comparisons (*p* < 0.05).

As opposed to MEP amplitude decline in the RS-baseline, the RS interacted with the LICI when combining the RS and the LICI paradigms, demonstrated as “Paradigm*Stimulus Order” interaction effect (*F*(3,56) = 4.12, *p* = 0.081 for the absolute MEPs and *F*(3,56) = 15.04, *p* = 0.006 for the normalized MEPs) (Fig. 1). This non-linear modulatory effect was observed as an increase in normalized MEP amplitudes during RS-LICI (Fig. 1a). *Post hoc* paired t-test revealed that the third MEP amplitude within RS-LICI trains was significantly higher than that in RS-baseline trains (*p* = 0.019 for the absolute MEPs and *p* < 0.001 for the normalized MEPs). No increase in the second and fourth MEP amplitudes was observed (*p* > 0.1). The Main effects of “Paradigm” and “Stimulus Order” on MEPs were also investigated (*F*(3,56) = 4.12, *p* = 0.082 (Paradigm) and *F*(3,56) = 3.10, *p* = 0.127 (Stimulus Order) for the absolute MEPs and, *F*(3,56) = 38.44, *p* = 0.001 (Paradigm) and *F*(3,56) = 5.32, *p* = 0.054 (Stimulus Order) for the normalized MEPs).

Interaction between the RS and the SICF were observed through a combined RS-SICF paradigm. Using the absolute MEPs illustrates the common offset added to the RS by SICF clearly. Main effect of “Paradigm” revealed that the RS-SICF paradigm increased the MEP amplitudes by a common offset (*F*(3,56) = 7.22, *p* = 0.031) for the absolute MEPs and *F*(3,56) = 8.34, *p* = 0.023 for the normalized MEPs), while the absolute RS effect remained unchanged (*p* > 0.5) (Fig. 1), as hypothesized. The main effect of “Stimulus Order” on MEPs was also demonstrated (*F*(3,56) = 16.62, *p* = 0.005 for the absolute MEPs and *F*(3,56) = 5.53, *p* = 0.048 for the normalized MEPs). *Post hoc* paired-samples t-test revealed the significant increase of the MEP amplitudes within the RS-SICF trains (*p* = 0.013, *p* = 0.044, *p* = 0.031 and, *p* = 0.031 for the absolute MEPs, in the first, second, third and, fourth stimulus, respectively and *p* = 0.050, *p* = 0.034, *p* = 0.030 for the normalized MEPs in the second, third and, fourth stimulus, respectively).

## Discussion

The findings from the present study suggest that combining the RS and LICI causes the RS to be overridden by the interaction with LICI, hence suggesting an interacting mechanism at the cortex between the two inhibitory phenomena. Since the ISI at which LICI occurs was 100 ms in our study, the time course at which LICI persists is over 100 ms following the first stimulus. On the other hand, the ISI between the consecutive pulses in the RS paradigm, 1 s, confirms its inhibitory action around 1 s. Hence, the relatively long time course of these two inhibitory phenomena raises the possibility of their interaction.

The RS is demonstrated by characteristic immediate drop in the MEP amplitude after the first stimulus, and a slight recovery of amplitudes may be observed towards the baseline^28^. The immediate drop is considered to occur to prevent meaningless movements by increase of the intracortical inhibition after a movement executed without prefrontal planning^17^. A previous study reported that in the active muscles the TMS-induced silent periods increase over time during the RS, suggesting that cortical inhibition is becoming stronger with repeated stimuli^19^. Hence, the suppression via cortical inhibition is strengthened after each repeated stimulus perhaps to balance the drive towards homeostatic baseline, which could be reflected as the observed gradual recovery in responses during RS, demonstrating an ongoing chance in the inhibition/excitation balance. In progressive myoclonus type 1 (EPM1) the inhibitory tonus is impaired^29^, and the observed abnormal RS is weaker^14^. As the inhibitory tonus is prevailing and saturated, disabling the maintained RS and enabling an immediate recovery in adolescent patients, the mechanism described above could be a viable explanation for the observed recovery. Although several mechanisms have been proposed, the underlying neurophysiologic basis of the RS has remained elusive^11^. The heightened amplitude of the initial response as compared to the consecutive responses can be explained by the arousal effect; an effect originated in the brainstem ascending reticular activation system (RAS) in response to novel stimuli^30,31^. A potential model explaining the attenuation of the consecutive responses is the Sharpening model. According to this model, the population size of the firing neurons is optimized to a maximal capacity of the target organ by a feedback loop as the stimulus is repeated^12,30,32^.

Another plausible underlying physiological mechanism might contribute to the increased activity of the GABAergic inhibitory system^19^, a mechanism that is mediating the LICI^22^. Apart from the production mechanisms of these two inhibitory phenomena, it has been suggested that they occur at different levels of the central nervous system. The RS is thought to occur at the thalamo-cortical level, while the LICI originates at the cortical cortical level^7,14,16,20^. Therefore, most likely, RS is ruled out by LICI at the cortex, tapping into the thalamo-cortical feedback loop that is activated by the RS. To elaborate on this, one possible explanation for the underpinning mechanism of the observed interaction between the RS and LIC is the activation of the thalamo-cortical feedback loop. We speculate that similar to the baseline measurement, the negative feedback from the cortical areas such as tightly interconnected sensory areas close by may increase the GABAergic intracortical inhibition after the first stimulus, which in turn causes the suppression of the second MEP amplitude. To restore the balance between the inhibitory and excitatory states of the motor network, the negative feedback loop weakens the thelamo-cortical inhibition and disinhibition dominates, following the second stimulus, resulting in a greater MEP during the third stimulus. During the third stimulus, the feedback loop is attenuated again, allowing for a greater response to occur. Higher response causes the enhancement of the function of the negative feedback loop following the third stimulus, resulting in suppression of the fourth response. Even though, our results cannot directly pin-point the thalamo-cortical pathway as the interaction locus for RS and LICI, this possibility cannot entirely be ruled out either. Animal studies have demonstrated activation of the inhibitory thalamic reticular nuclei following stimulation of the cerebral cortex^33,34^. This activation can result in inhibition of the cerebro-thalamo-cortical pathway. It has been suggested that the conditioning stimulus of the LICI may affect the thalamo-cortical pathway at the thalamus^35^. Therefore, there is a possibility that the interaction site is at the subcortical level.

We observed that I1-wave timed SICF facilitated all MEP responses without modulating the absolute RS, meaning the inhibitory effect appeared not to affect the I1-wave SICF interaction. There is growing evidence on the stability of I1-wave, making it implausible to be modulated by inhibitory and excitatory mechanisms^23,24,36^. Setting the time interval between the two consecutive pulses in SICF in such manner that I1-wave is targeted can enhance the effects of TMS by influencing the absolute momentary motor cortical activation, probably via synchronization of I2-wave in conditioning pulse with I1-wave in test pulse. Facilitatory effect of the I1-wave timed paired-pulse TMS has been recently demonstrated with biphasic TMS waveform^10^. In accordance with these data, our finding also suggests facilitation effect observed, using I-wave-timed paired-pulse TMS, where the inhibitory RS remains unchanged, i.e., the observed effect in the normalized MEPs was explained fully by the common offset added by the SICF to all absolute MEP responses in the RS paradigm.

Detrimental parameters that need to be taken into account to optimize the probability of the occurrence of the required paradigms, e.g. the RS and LICI, are the intensity, the location of the stimulus and the direction of the induced current, and ITI^28,37–40^. In the current study, ITI of 17 s was employed in all RS paradigms, since it has been depicted that RS effect is more pronounced at this ITI, probably due to more time to recover. No carry-over effects from trial to trial have been observed at this ITI^28^. Similarly, we optimized the parameters for LICI and SICF protocols to optimize the inhibitory and facilitatory effects confirmed as successful by our baseline measurements^9,10,19^. Altering the applied parameters for the SICF and LICI could have altered the results.

In conclusion, in agreement with our hypothesis, the SICF did not interact with the RS, but facilitated all responses in the RS train. Again, in agreement with our hypothesis, the LICI demonstrated an interaction effect with RS, exhibiting a non-linear interaction partly reducing the common RS effect potentially interfering with the RS at the cortical level.

## Methods

### Subjects

Eight healthy right-handed volunteers with no history of neurological disorders were recruited in this study (6 males, aged 22–42 years). Written informed consents were obtained from all participants. The study was approved by the research ethics committee of the Kuopio University Hospital (256/2017). The study was performed in accordance with the Declaration of Helsinki abiding the safety guidelines for TMS applications^41^.

### Transcranial magnetic stimulation (TMS)

**Figure 2:**
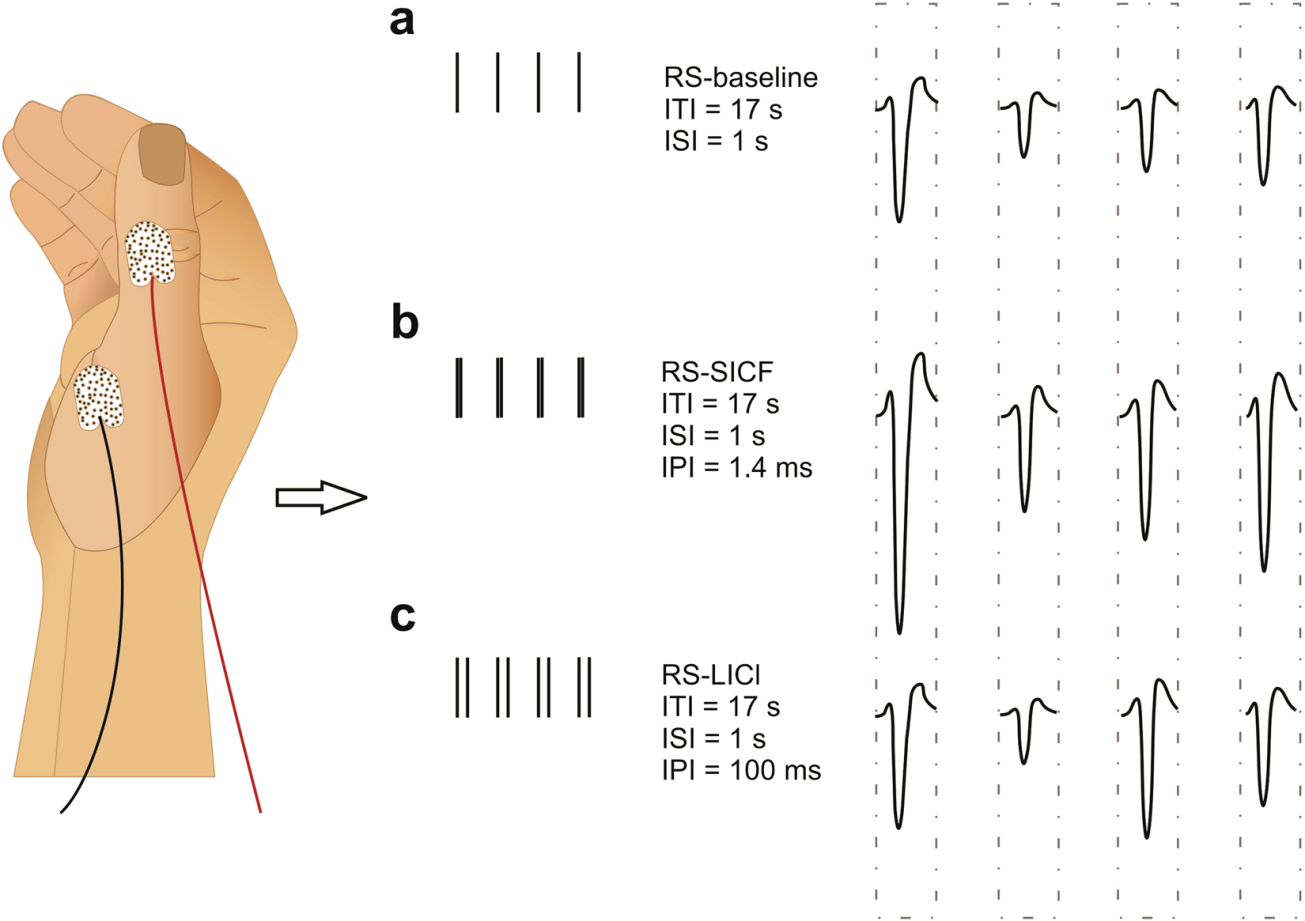
Schematic figure of three TMS stimulation paradigms to study RS via MEPs of target APB muscle. (**a**) In baseline paradigm for RS, twenty trains of four single biphasic pulses were applied. (**b**) In combining SICF and RS (RS-SICF), twenty trains of four biphasic paired pulses at an IPI of 1.4 ms were used to induce the SICF. (**c**) In combining LICI and RS (RS-LICI), twenty trains of four paired pulses with IPI of 100 ms were utilized to induce LICI. ITI of 17 s and ISI of 1 s were utilized in all paradigms.

The measurements were performed using a customized NBS System 4.3 (Nexstim Plc., Helsinki, Finland) with an air-cooled figure-of-eight coil. Prior to the study, structural T1-weighted magnetic resonance images (MRIs) were acquired with a 3T MRI scanner (Philips Achieva 3.0T TX, Philips, The Netherlands). MRI data were further utilized in neuronavigation. The initial step in stimulation procedure was to determine the abductor pollicis brevis (APB) muscle “hotspot”, i.e. the cortical site capable of eliciting the maximal contralateral muscle response with minimal stimulator output intensity. Once the hotspot was identified, the resting motor threshold (rMT) was determined using system-integrated iterative threshold assessment tool^10^. The subsequent TMS stimuli were administrated over the APB hotspot at an intensity of 120% rMT during three different paradigms used during this study: RS-baseline, RS-SICF and RS-LICI, utilizing the biphasic waveform (Fig. 2). All RS paradigms included twenty trains of four trials. Four trials have been demonstrated to evoke a reliable RS effect^14,28^. The trials consisted of the stimuli either from a single-pulse form (RS-baseline) or a paired-pulse form (RS-SICF and RS-LICI). In RS-SICF a suprathreshold pulse was followed by a subthreshold one at an inter-pulse interval (IPI) of 1.4 ms^10^, whereas in RS-LICI, a conditioning suprathreshold pulse was delivered 100 ms before a suprathreshold test pulse^42^. An inter-stimulus interval (ISI) of 1 s within the trials and inter-train interval (ITI) of 17 s maintained throughout all the paradigms^28^. For confirming the induction of the LICI, baseline measurements were also conducted prior to the RS experiments applying 20 trials of paired pulses at 120% rMT at IPI of 100 ms at the ITI of about 5 s and comparing the first response amplitude to the second one in the stimulus pairs^43^. The occurrence of SICF was verified at the first stimuli of the RS-SICF protocol by comparing those to the first stimuli of the RS-baseline protocol.

### Electromyography (EMG)

EMG responses, i.e. MEPs, were recorded via an integrated EMG system at the sampling frequency of 3 kHz. A pair of disposable Ag-Cl electrodes were utilized with the active electrode placed over the belly of the APB muscle and a reference electrode placed over the joint distal to the active electrode (Fig. 2). The MEPs were recorded by triggering the EMG signal with TMS, and were processed offline in Matlab (version R2017b, MathWorks Inc., Natick, MA, USA). The timing and intensity of the stimuli within the protocols was set with the integrated navigation and recording system software at a millisecond precision. The IPI for the paired-pulses can be adjusted at a 0.1 ms precision. MEPs occurring in the resting muscles with peak-to-peak amplitude greater than 50 μV, were considered as MEPs.

### Statistical analysis

MEP data in each paradigm were first averaged over all the trains on the basis of their stimulus order in a train, for each subject. First, to assess the RS effect in the baseline condition, repeated measures ANOVA with “Stimulus Order” within a train (first, second, third and, fourth) as fixed effect was applied. Also, to evaluate the inhibition effect in LICI baseline, Wilcoxon signed-rank test was conducted to compare the test pulses against the conditioning ones. A two-way repeated measures ANOVA with “Paradigm” (RS-baseline, RS-LICI and, RS-SICF) and “Stimulus order” (first, second, third and, fourth) as within-subject factors was employed to evaluate the influence of the paradigms and stimulus order as main effects, as well as their interaction effect. Further, *Post-hoc* tests were performed to assess the effect of the paradigms on MEPs at each stimulus order. All tests were conducted on both absolute and normalized MEPs. Normalization was performed by dividing all average MEP amplitudes within the trials with the average amplitude of the first MEP. *Post-hoc* comparisons were conducted using paired *t*-test with Bonferroni correction. A *p*-value of <0.05 indicated statistical significance. Statistical analyses were conducted using SPSS (v. 25.0, SPSS Inc., IBM Company, Armonk, NY, USA) and Matlab (version R2017b, MathWorks Inc., Natick, MA, USA).

## Data Availability

The datasets generated and analyzed during the current study are available from the corresponding author on reasonable request.
